# Analysis of food supplements and sports foods consumption patterns among a sample of gym-goers in Portugal

**DOI:** 10.1080/15502783.2024.2388077

**Published:** 2024-08-08

**Authors:** Sofia Lopes, Madalena Cunha, João Guilherme Costa, Cíntia Ferreira-Pêgo

**Affiliations:** a CBIOS – Universidade Lusófona’s Research Center for Biosciences and Health Technologies, Lisbon, Portugal; b Universidade Lusófona, School of Health Sciences and Technologies, Lisbon, Portugal

**Keywords:** Food supplements, Dietary supplements, Sports foods, Exercise, Gym-goers, Nutrition

## Abstract

**Background:**

Gym-goers usually seek methods to improve performance, muscle gain, and overall health. One of the main strategies is including food supplements (FS) into their routine as aids to enhance their athletic capabilities and satisfy their nutritional needs. Thus, this study aimed to investigate and characterize the main FS and Sports Foods (SF) currently consumed, as well as the main reasons for their use and the source of advice in a group of gym-goers in the Lisbon Metropolitan Area (Portugal).

**Methods:**

A cross-sectional study was conducted, including 303 gym-goers from Lisbon, Portugal, who were 133 women and 170 males (30.8 ± 12.9 years old). Face-to-face interviews were used by qualified researchers to gather data.

**Results:**

Most of the interviewed athletes (71.95 %) took FS/SF, being men the main consumers. On average, 1.59 supplements were consumed per athlete. Logistic regression models indicated significant associations between age, gender, and motivations for gym attendance. While men and younger groups attended mainly for hypertrophy, women and older groups were focused on well-being. Protein (59.17 %) was the most used FS/SF, followed by creatine (41.28 %) and multivitamins (27.06 %). Men and younger individuals preferred protein and creatine, while older individuals focused more on specific vitamins and minerals. Women seemed to prefer L-carnitine and protein yogurts. Main sources of information included the internet, friends, and dietitians with notable gender and age-based preferences. Online stores were the main place of purchase. Monthly expenditures on FS/SF were not significantly affected by age or gender, but motivations for use had an influence.

**Conclusion:**

Most of the athletes interviewed took FS/SF, being men the major consumers. Protein was the principal FS/SF used, with online stores being the main place of purchase and the internet the primary source of information. Age and gender were key factors in adopted training, in the FS/SF chosen, and in the source of information selected. It is crucial that health professionals take primary responsibility for providing this guidance.

## 1. Introduction

Physical exercise plays a fundamental role in the health and well-being [1]. It contributes to the improvement of some esthetical components [2,3] and also serves as a preventive measure against several diseases [1]. As a result, there has been an exponential growth in the number of individuals seeking to attend gyms [4].

Nutrition is one of the key elements for athletic performance [5]. Appropriate meals taken at optimal times provide the essential nutrients to support physical activity, enhance performance, and reduce fatigue [5–7]. However, exercise can change the body’s nutritional requirements by increasing metabolic rates, which raises the amount of energy expended [5]. This leads to necessary adjustments in calorie intake to avoid deficiencies and to support the body’s recovery [5]. There are several essential components for athletes such as protein [5], which is crucial for muscle growth and repair [8]; adequate hydration, to maintain electrolyte balance and achieve optimal performance [9]; and carbohydrates, which are important for muscle glycogen replacement [10].

Throughout history, athletes have consistently sought ways to enhance their physical condition, finding aids for performance, speed, and strength in specialized diets or products [11]. In the past, these practices included the consumption of lion’s heart and deer’s liver [11]. However, with the increase in knowledge about the physiology of exercise, specific sports-related food supplements (FS) also known as Dietary Supplements, Sports Foods (SF), and/or other ergogenic aids have become integral parts of athletes’ routines [11–13], making it easier and simpler to reach their nutritional requirements [5,11]. Currently, athletes constitute a significant focus for the supplement industry, due to their large and diverse consumption of FS and SF [6,14].

Despite the lack of evidence regarding the effectiveness and safety of these products, sportspeople have various incentives for incorporating supplements into their routines [15,16]. Often, some of these motivations are acquired in the gym environment itself, either from other athletes or even by professionals [15,16]. These incentives include improving recovery [6,7,17] through the use of carbohydrates [10], promoting general health [6,7,17] with multivitamins [18], enhancing performance [6,7,12,19] associated with the use of substances like caffeine [18,20] and creatine [18,21], improving muscle growth [6,7,12] through the consumption of amino acids and proteins [8] and preventing or treating injuries [6], by including supplements like glucosamine [22] in their diets.

Nevertheless, for recreational athletes, supplementation should only be considered if the athlete is not following a well-balanced diet [6,7], is on a restricted diet, experiencing increased nutrient and energy needs, or experiences discomfort with specific foods [6,23]. From a clinical perspective, evidence suggests that FS rarely provided benefits compared to a proper diet (except for specific groups, such as pregnant women, for example) [23]. The improper use of these products can have negative effects on athletes’ performance and health, making professional guidance essential. The most frequently reported adverse effects include palpitations, chest pain, and tachycardia, often linked with the use of weight loss promoters and SFs intended for muscle growth [24]. The risk of adverse reactions rises when multiple FS are consumed simultaneously since some substances can have additive or synergistic effects [25]. Many consumers perceive FS as “miraculous” products, not considering the possible adverse effects tied to them [26,27]. The lack of knowledge among supplement consumers often stems from a misunderstanding of the term itself, mistakenly equating FS/SF as natural products [25].

The current version of Portuguese Decree of Law nº 136/2003 [28] defines FS as “Foodstuffs intended to supplement a normal diet and which are concentrated sources of nutrients (e.g. vitamins or minerals) or other substances with a nutritional or physiological effect, alone or in combination, marketed in dosage form: capsules, tablets and similar forms, sachets of powder, ampoules of liquids, dosing bottles and other similar forms, oral dosage forms, liquids and powders intended to be taken in single units of reduced quantity” [29]. On the other hand, SF is categorized as conventional foodstuffs [30]. However, this type of product has characteristics that can hinder its classification as a standard foodstuff, especially when compared to FS, leading it to be classified as a borderline product [30]. To classify a product as FS, certain criteria need to be met. These include the presentation of the product in a dosed form (tablets, capsules, sachets, and others similar to pharmacological ones, including measuring spoons, cups, or dispensers) [30,31]. Furthermore, the product should be intended for consumption in measured units of reduced quantity (maximum 25 g or 25 mL) [30,31] and the total energy value of the daily intake should not exceed 50 kcal [30]. Products that do not meet the specified requirements fall under the category of SF [30,31]. Hence, it is up to the manufacturer to determine the classification framework for the product they intend to introduce on the market [30,31].

As the FS market expands, there is increasing evidence suggesting potential risks associated with these products. These hazards stem from factors like inadequate quality control, inappropriate storage conditions, or the intentional addition of unauthorized components aimed to increase the product’s efficacy [11,32]. Consequently, there exists a valid concern regarding intrinsic toxicity and the risk of contamination or adulteration of FS, which could pose significant risks to human health [11,32]. Contaminants and adulterants in FS may range from heavy metals to undeclared and illegal substances [11,32,33].

Hence the novelty of the present work resides in the increasing use of FS and SF in a sports context [30,31], being crucial to view them as a strategy for specific nutrient requirements [30,31]. Nevertheless, the information gap about its real consumption, the type of products consumed together with their constraints, would determine the necessity of exploring and updating this topic. Under this context, this work aimed to investigate and characterize the main FS and SF currently consumed, as well as the main reasons for their use and the source of advice in a group of gym-goers in Lisbon, Portugal.

## 2. Materials and methods

### 2.1. Study design and population

The present cross-sectional study was conducted in several gyms in the Lisbon Metropolitan Area, Portugal, in a face-to-face interview and occurred randomly between March and May 2023. The inclusion criteria were being gym-goers (all individuals who attend and exercise at the gym), aged 18 years or older, being Portuguese speaking, and residing in the Lisbon metropolitan area. On the other hand, the non-inclusion criteria were having health conditions that could affect the findings, pregnancy or breastfeeding, being federated athletes, living abroad, or not meeting any of the inclusion criteria. The final sample included 303 people of both biological sexes, with ages ranging from 18 to 76 (30.82 ± 12.84) years old.

The formula used to determine the sample size was as follows: *n* = z^2^ * p (1-p)/W^2^ [34,35], where n is the estimated sample size, z is the normal distribution (defined as 1.96 for studies with a 95 % confidence level), p stands for the estimated number of gym members in Portugal and W is the margin of error of the study (6 %). The p was obtained from the most recent data from the Fitness Barometer in Portugal (2022), developed by Portugal Ativo and the Center for Economic and Institutional Studies of the Autonomous University of Lisbon, which stated the estimated number of active gym members in Portugal in 2022 was 691,656 [4]. Since there is no official data for the Lisbon region, we kept the national value of p, but the margin of error was increased to 6 %. The final sample size calculated was 267 participants.

All ethical guidelines developed for studies involving humans were followed during the procedures, as per the Helsinki Declaration [36]. All participants gave their oral consent before inclusion in the project. The Ethics Committee of the School of Health Sciences and Technologies at Lusófona University already gave its approval to the project (CE.ECTS/P12–23).

### 2.2. Participants’ characterization and instruments

The data collection tool was a questionnaire adapted from the works of Lacerda et al.. (2015) [37], Jawadi et al. [38], and Ruano et al. [14]. The questionnaire was divided into three main sections and included 28 questions:

1. general characterization and lifestyle, which included age, gender, education, employment, place of residence, marital status, monthly incomes, health status, use of medications, and smoking habits. Body mass and height were self-reported variables used to calculate Body Mass Index (BMI) according to Quetelet’s formula [body mass (kg)/height squared (m^2^)] [39].
2. the second section consisted of the collection of exercise data, and it requested gym membership duration, frequency, duration, level, and type of exercise in and out of the gym, as well as training goals.
3. the last section was about the FS and SF consumed (currently or/and in the previous year), in response to this question, if the respondent acknowledged using FS or SF, they should provide a comprehensive list of the top five most consumed FS/SF, along with the dosage and brand. In this section, were also included questions about the reasons for use, sources of information, level of satisfaction, and place of purchase. For this study, we used the definition of FS found in Directive 2002/46/CE and Decree-Law nº 118/2015 [28] (“Foodstuffs intended to supplement the typical diet, consisting of a concentrated source of a nutrient or of other substances that have a nutritional or physiological effect, in a simple or combined form, commercialized in dosed formulas, capsules, tablets, pills and other similar forms, bags of powder, vials of liquid, dropper bottles and other similar forms of liquids and powders, which is taken in small, quantified amounts”).

### 2.3. Statistical analysis

Statistical analysis was performed using the Statistical Package for Social Sciences (IBM SPSS) version 22.0 (SPPS Inc., Chicago, IL). Variables normality was tested, parametric tests were used when the sample presented a normal distribution, and non-parametric tests when the sample presented a non-normal distribution. Based on the type of variable, distinct tests were applied. The Kolmogorov-Smirnov normality test was used when *n* > 50 and the Shapiro-Wilk normality test when *n* < 50. Nominal variables were shown as percentages (frequencies), and continuous variables as mean (standard deviation, SD) or median (interquartile range, IQR), depending on the type of variable. For the comparison between two categorical variables, the Chi-squared, Fisher’s Exact, and Monte Carlo tests were used, as appropriate. On the other hand, between a categorical variable and a scale variable, the Student’s t-test, and Mann-Whitney U test were used. The consumption of FS and other variables that were relevant to analyze with the respective risk analysis through binary logistic regression (with a 95 % confidence interval) were subjected to Pearson’s correlation analysis to determine whether there was any strong interrelationship between them. All statistical tests were two-tailed, and the significance level was set at *p* < 0.05.

## 3. Results

Of the 303 survey participants, 85 (28.05 %) were not consuming FS, whereas 218 (71.95 %) were supplement consumers. Most respondents were male (56.11 %), employed (54.10 %), and single (77.90 %). At the time of the study, most of the sample were nonsmokers (64.69 %) and did not report any chronic conditions (91.70 %). The general characteristics of the study population are shown in Table 1. The mean age of the total population was 30.82 ± 12.84 years old, the median height was 1.72 ± 0.13 m, the median body mass was 70.00 ± 18.00 kg, and the median BMI was 23.51 ± 4.04 kg/m^2^. Supplement consumers were mostly men (*p* = 0.003) and showed significantly high values of height and body mass. No statistically significant differences (*p* = 0.589) in ages were found between men (M_e_ = 30.01 years old) and women (M_e_ = 31.85 years old) [**data not shown**].

**Table 1.** The sociodemographic and lifestyle characteristics of all participants, food supplements and sports foods consumers, and non-consumers.

	Total Population (*n* = 303)	Supplement Non-Consumers (*n* = 85)	Supplement Consumers (*n* = 218)	p-value
**Gender, % (n)**				
Male	56.11 (170)	42.35 (36)	61.47 (134)	0.003^b^
Female	43.89 (133)	57.65 (49)	38.53 (84)	
**Age, % (n)**				
18 – 25 years old	48.85 (148)	47.06 (40)	49.54 (108)	0.125 ^b^
26 – 40 years old	29.04 (88)	23.53 (20)	31.19 (68)	
41 – 80 years old	22.11 (67)	29.41 (25)	19.27 (42)	
**Height, m**	1.72 (0.13)	1.68 (0.13)	1.73 (0.15)	0.008^a^
**Mass, kg**	70.00 (18.00)	66.00 (18)	72.00 (17.25)	0.006^a^
**BMI, kg/m**^**2**^	23.51 (4.04)	23.31 (4.33)	23.53 (3.89)	0.253^a^
**Monthly Family Income, % (n)**				
Until 1000 €	8.58 (26)	8.24 (7)	8.72 (19)	0.177^b^
1000 € - 3000 €	53.14 (161)	56.47 (43)	51.83 (113)	
> 3000 €	31.68 (96)	24.71 (21)	34.40 (75)	
**Educational qualifications, % (n)**				
Undergraduate	45.21 (137)	45.88 (39)	44.95 (98)	0.884 ^b^
Graduate	54.79 (166)	54.12 (44)	55.05 (120)	
**Smoking Status, % (n)**				
Non-Smoker	64.69 (196)	61.18 (45)	66.06 (144)	0.658^b^
Ex-Smoker	17.16 (45)	17.65 (15)	16.97 (37)	
Smoker	18.15 (46)	21.18 (18)	16.97 (37)	
**Gym Membership Duration, % (n)**				
< 1 year	31.35 (95)	41.18 (35)	27.52 (47)	0.085^b^
1 - 2 years	24.09 (73)	24.71 (21)	23.85 (45)	
2 - 5 years	24.09 (73)	17.65 (15)	26.61 (48)	
> 5 years	20.47 (49)	16.46 (14)	22.02 (43)	

Data are expressed as a percentage (n) or median (IQR) for categorical or continuous variables. P-values for group comparisons were assessed by ^a^ ‘Mann-Whitney U Test or ^b^ Chi-squared, as appropriate. Abbreviations: BMI, Body Mass Index.

The outcomes of our study show that younger age groups go to the gym seeking to hypertrophy and improve performance (*p* < 0.001), while older individuals attend more with the purpose of physical and psychological well-being (*p* < 0.001). Men tend to start going to the gym with the goal of hypertrophy and performance improvement (*p* < 0.001), but women tend to start with the purpose of well-being (*p* < 0.05) and weight loss (*p* < 0.001). Table 2 also presents a predictive logistic regression model to identify the age and gender of a population’s motivation for physical activity. According to the findings, younger age groups (18 to 25 years old) appear to have a 7,680 times higher probability of going to the gym for hypertrophy, whereas older age groups (41 to 80 years old) have an 8,458 times higher probability of going seeking for well-being. In terms of gender, women are 2,738 times more likely than men to go to the gym for weight loss, while men are 2,949 times more likely to go for muscle gain (Table 2).

**Table 2.** Predictive logistic regression model for determining the age and gender of the general population fitness motivation.

	Total Population (*n* = 303)	Age						Gender				
Fitness Motivation		18-25 years old (*n* = 148)	26-40 years old (*n* = 88)	41-80 years old (*n* = 67)	p-value^a^	OR (95 % CI)		Male, M (*n* = 170)	Female, F (*n* = 133)	p-value^a^	OR (95 % CI)	
Weight Management gym goal	23.10 (70)	19.60 (29)	29.55 (26)	22.39 (15)	0.212			15.29 (26)	33.08 (50)	**<0.001**	**M**	0.365 (0.210 – 0.634)
											**F**	2.738 (1.576 – 4.756)
Hypertrophy gym goal	46.21 (140)	60.14 (89)	45.45 (41)	16.42 (11)	**<0.001**	**18-25**	7.680 (3.718 -15.861)	57.65 (98)	31.58 (42)	**<0.001**	**M**	2.949 (1.833 – 4.745)
						**26-40**	4.242 (1.963 -9.169)				**F**	0.339 (0.211 – 0.546)
						**41-80**	0.130 (0.063 – 0.269)					
Well-Being gym goal	59.41 (180)	43.24 (64)	65.91 (48)	86.57 (48)	**<0.001**	**18-25**	0.118 (0.055 -0.256)	52.35 (89)	68.42 (91)	**0.005**	**M**	0.507 (0.316 – 0.814)
						**26-40**	0.300 (0.131 -0.687)				**F**	1.972 (1.228 – 3.166)
						**41-80**	8.458 (3.901 - 18.338)					
Performance gym goal	16.83 (51)	18.24 (27)	22.73 (20)	5.97 (4)	**0.018**	**18-25**	3.514 (1.178 -10.488)	22.35 (38)	9.77 (13)	**0.004**	**M**	2.657 (1.351 – 5.227)
						**26-40**	4.632 (1.501 -14.296)				**F**	0.376 (0.191 – 0.740)
						**41-80**	0.285 (0.095 –0.849)					

Data are expressed as percentages (n). ^a^ Statistical significance *p* < 0.05. P-values for group comparisons were assessed by the Chi-squared test.

**Figure 1.**
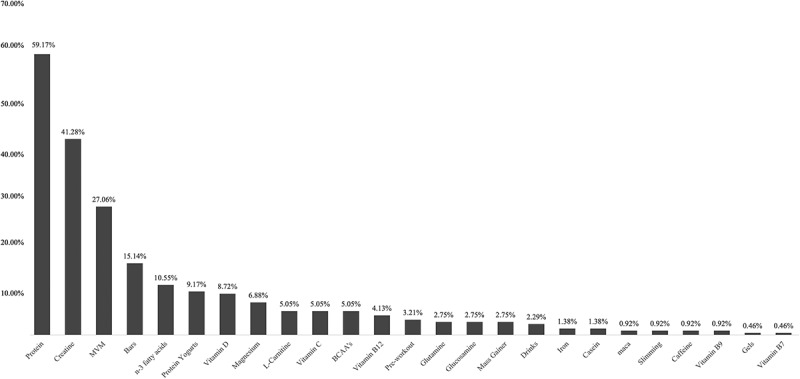
Most consumed food supplements and sports foods, per category, by gym-goers; BCAA: branched-chain amino acids; MVM: multivitamin and/or minerals supplements.

**Figure 2.**
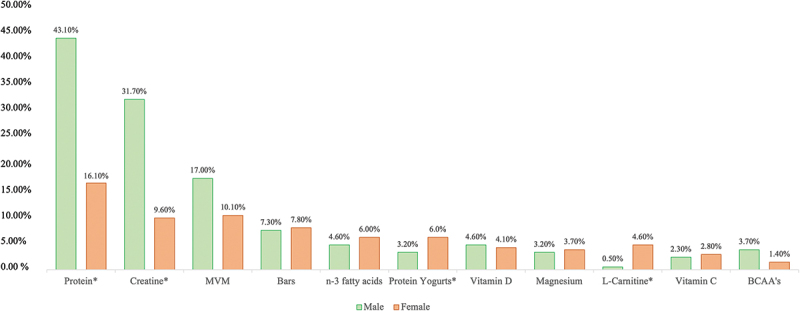
Most used food supplements and sports foods, per sex by gym-goers; BCAA: branched-chain amino acids; MVM: multivitamin and/or minerals supplements. * statistical significance *p* < 0.05.

According to our data, the FS and SF more popularly ingested by gym-goers were protein powders (59.17 %), followed by creatine (41.28 %), multivitamins and/or minerals (MVM) (27.06 %), sport bars (15.14 %), *n*-3 fatty acids (10.55 %), protein yogurts (9.17 %), Vitamin D (8.72 %), Magnesium (6.88 %), L-Carnitine (5.05 %), Vitamin C (5.05 %) and Branched-Chain Amino Acids (BCAA’s) (5.05 %) (Figure 1). Among the 218 participants, 16 individuals (7.30 %) reported simultaneous consumption of five FS. The combinations most frequently consumed were protein powders and creatine (32.11 %), protein powders and MVM (14.68 %), and creatine with MVM (12.39 %) **[data not shown]**. In comparison to women, a higher percentage of men appear to use protein, creatine, MVM, vitamin D, and BCAA’s. Women appear to prefer sports bars, *n*-3 fatty acids, protein yogurts, magnesium, L-carnitine, and vitamin C (Figure 2).

**Figure 3.**
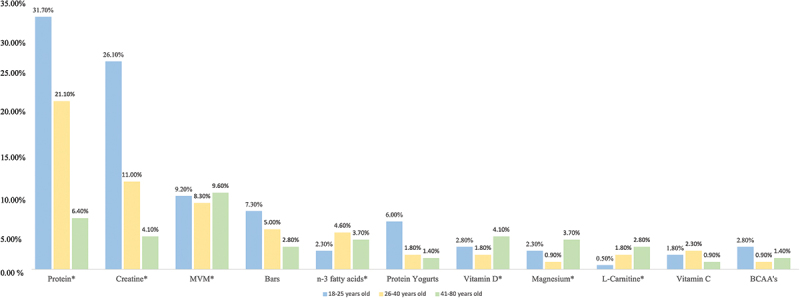
Most used food supplements and sports foods, per age by gym-goers; BCAA: branched-chain amino acids; MVM: multivitamin and/or minerals supplements. * Statistical significance *p* < 0.05.

Regarding the FS most consumed by age, according to our data, younger age groups appear to consume more protein, creatine, sports bars, protein yogurts, and BCAAs. In contrast, it appears that a higher percentage of older people use MVM, vitamin D, magnesium, and L-carnitine (Figure 3).

Referring to Table 3, which presents the results of the predictive logistic regression model, it is observed that men were 3.290 times more likely than women to use protein powders and 3.185 times more likely to ingest creatine. In contrast, women are 17.973 times more likely than men to take L-carnitine and have a 3.322 times higher probability of consuming protein yogurt (Table 3). Moreover, 5 out of the 9 FS shown were associated with older age categories: MVM (OR = 4.400; CI: 2.026–9.556; p = 0.001), *n*-3 fatty acids (OR = 4.847; CI: 1.485–15.817; p = 0.014), Vitamin D (OR = 4.636; IC: 1.536–13.999; p = 0.005), Magnesium (OR = 4.847; CI: 1.485–15.817; p = 0.03) and L-Carnitine (OR = 17.847; CI: 2.076–153.161; p = 0.003). Creatine and protein powders were the only two products associated with the younger categories (Table 3).

**Table 3.** Predictive logistic regression model for determining the age and gender of food supplements and sports foods consumers.

	Age						Sex				
FS Consumed	18-25 years old (*n* = 108)	26-40 years old (*n *= 68)	41-80 years old (*n* = 42)	p-value	OR (95 % CI)		Male, M (*n* = 134)	Female, F (*n* = 84)	p-value	OR (95 % CI)	
Protein	63.89 (69)	67.65 (44)	33.33 (14)	**0.001**^a^	**18-25**	3.538 (1.668 - 7.507)	70.15 (94)	41.67 (35)	**< 0.001**^a^	**M**	3.290 (1.860 – 5.818)
					**26-40**	4.182 (1.845 - 9.479)				**F**	0.304 (0.172 – 0.538)
					**41-80**	0.283 (0.133 - 0.600)					
Creatine	52.78 (52)	35.29 (24)	21.43 (9)	**0.001**^a^	**18-25**	4.098 (1.790 - 9.381)	51.49 (69)	25.00 (21)	**< 0.001**^a^	**M**	3.185 (1.750 – 5.796)
					**26-40**	2.000 (0.822 - 4.866)				**F**	0.314 (0.173 – 0.572)
					**41-80**	0.244 (0.107 - 0.559)					
MVM	18.52 (20)	26.47 (18)	50.00 (21)	**0.001**^a^	**18-25**	0.227 (0.105 -0.494)	27.61 (37)	26.19 (22)	0.818 ^a^		
					**26-40**	0.360 (0.160 - 0.809)					
					**41-80**	4.400 (2.026 - 9.556)					
*n*-3 fatty acids	4.63 (5)	14.71 (10)	19.05 (8)	**0.014**^a^	**18-25**	0.206 (0.063 – 0.673)	7.46 (10)	15.48 (13)	0.061 ^a^		
					**26-40**	0.733 (0.264 – 2.035)					
					**41-80**	4.847 (1.485 – 15.817)					
Sport Bars	14.82 (16)	16.18 (11)	14.29 (6)	0.956 ^a^			11.94 (16)	20.24 (17)	0.096 ^a^		
Protein Yogurts	12.03 (13)	5.88 (4)	7.14 (3)	0.341 ^a^			5.22 (7)	15.48 (13)	**0.011**^a^	**M**	0.301 (0.115 – 0.789)
										**F**	3.322 (1.267 – 8.708)
Vitamin D	5.56 (6)	5.88 (4)	21.43 (9)	**0.005**^a^	**18-25**	0.216 (0.071 – 0.651)	7.46 (10)	10.71 (9)	0.407 ^a^		
					**26-40**	0.229 (0.066 – 0.800)					
					**41-80**	4.636 (1.536 – 13.999)					
Magnesium	4.63 (5)	2.94 (2)	19.05 (8)	**0.003**^b^	**18-25**	0.206 (0.063 – 0.673)	5.22 (7)	9.52 (8)	0.222 ^a^		
					**26-40**	0.129 (0.026 – 0.640)					
					**41-80**	4.847 (1.485 – 15.817)					
L-Carnitine	0.93 (1)	5.88 (4)	14.29 (6)	**0.003**^b^	**18-25**	0.056 (0.007 – 0.482)	0.75 (1)	11.90 (10)	**<0.001**^c^	**M**	0.056 (0.007 – 0.443)
					**26-40**	0.375 (0.099 – 1.417)				**F**	17.973 (2.256 – 143.173)
					**41-80**	17.847 (2.076 – 153.161)					
Vitamin C	3.70 (4)	7.35 (5)	4.76 (2)	0.504 ^b^			3.73 (5)	7.14 (6)	0.343 ^b^		
BCAA’s	5.55 (6)	2.94 (2)	7.14 (3)	0.666 ^b^			5.97 (8)	3.57 (3)	0.536^b^		

Data are expressed as a percentage (n) for categorical variables. P-values for group comparisons were assessed by ^a^ Chi-squared, ^b^ Monte Carlo’s, or ^c^ Fisher’s exact tests. Abbreviations: FS, Food Supplements; MVM, Multivitamin and/or minerals supplements.

**Figure 4.**
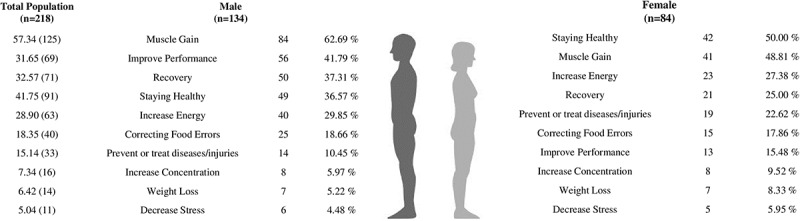
Reason for use referred to food supplements and sports foods consumers by sex.

**Figure 5.**
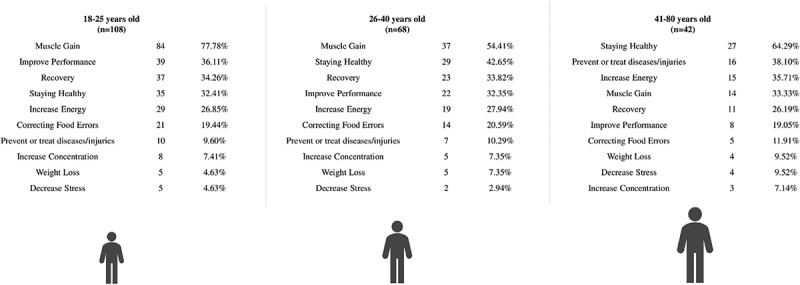
Reasons for supplement use referred by food supplements and sports foods consumers by age.

The most widely referenced reasons for consuming FS were muscle gain (57.34 %), staying healthy (41.75 %), faster recuperation (32.57 %), and increased sports performance (31.65 %) (Figure 4). The most often mentioned reasons for males were muscle gain (62.69 %), enhancing performance (41.79 %), and recovery (37.31 %). Women cited remaining healthy (50.00 %), muscle gain (48.81 %), and increasing energy (27.38 %) as primary motivations (Figure 4). When comparing age groups, younger volunteers cited muscle gain (77.78 %), improved performance (36.11 %), and recovery (34.26 %) as reasons for beginning FS use, while older volunteers cited staying healthy (64.29 %), preventing, or treating diseases/injuries (38.10 %), and energy boost (35.71 %) (Figure 5). In general, most of the participants (54.50 %) reported that the use of these FS matched their expectations.

Regarding the categorization of the acquisition place of the FS consumers by age and sex, 125 participants of the total sample (57.34 %) reported buying their FS at online stores, with supermarkets as the second option (32.57 %). Younger people seem to prefer to purchase their FS at online stores (62.04 % *vs*. 38.10 %, *p* = 0.019), while the older ones seem significantly to prefer pharmacies (*p* = 0.010) (Table 4). Men preferred to acquire FS through the Internet (66.42 % *vs*. 42.86 %, *p* < 0.05), while women were more likely to buy them often in pharmacies (6.72 % *vs*. 26.19 %, *p* < 0.001) and herbal stores (3.73 % *vs*. 11.90 %, *p* < 0.05) (Table 4).

**Table 4.** Categorization of the acquisition place by food supplements and sports foods consumers by age and sex.

	Total Population (*n* = 218)	Age				Sex		
Acquisition place		18-25 years old (*n *= 108)	26-40 years old (*n* = 68)	41-80 years old (*n* = 42)	p-value	Male (*n* = 134)	Female (*n* = 84)	p-value
Pharmacy	14.22 (31)	12.04 (13)	8.82 (6)	28.57 (12)	**0.010**^a^	6.72 (9)	26.19 (22)	**< 0.001**^a^
Parapharmacy	3.67 (8)	1.85 (2)	5.88 (4)	4.76 (2)	0.408^b^	3.73 (5)	3.57 (3)	1.000^c^
Supermarket	32.57 (71)	31.48 (34)	29.41 (20)	40.48 (17)	0.458^a^	29.10 (39)	38.10 (32)	0.168^a^
Online	57.34 (125)	62.04 (67)	61.77 (42)	38.10 (16)	**0.019**^a^	66.42 (89)	42.86 (36)	**0.001**^a^
Herbal Store	6.88 (15)	4.63 (5)	5.88 (4)	14.29 (6)	0.091^b^	3.73 (5)	11.90 (10)	**0.020**^a^
Supplement store	15.60 (34)	18.52 (20)	10.29 (7)	16.67 (7)	0.335^a^	17.91 (24)	11.90 (10)	0.234^a^

Data are expressed as a percentage (n) for categorical variables. P-values for group comparisons were assessed by ^a^ Chi-squared Test, ^b^ Monte Carlo’s Test, or ^c^ Fisher’s exact test, as appropriate.

Consumers of FS most frequently referred Internet (42.20 %), friends (26.61 %), Registered Dietitians (25.23 %), personal trainers (20.64 %), and Doctors (13.76 %) as sources of information. Women significantly preferred medical experts to get information (20.24 % *vs*. 9.70 %, *p* = 0.028), whereas males preferred personal trainers (26.87 % *vs*. 10.71 %, *p* = 0.004). It was also shown that the older group significantly referred to medical experts (*p* = 0.037) and themselves (*p* = 0.034) as sources of information (Table 5). A substantial percentage (56.90 %) of supplement consumers claimed that they were well-informed about FS usage.

**Table 5.** Sources of information referred to food supplements and sports foods consumers by age and sex.

	Total Population (*n* = 218)	Age				Sex		
References		18-25 years old (*n* = 108)	26-40 years old (*n* = 68)	41-80 years old (*n* = 42)	p-value	Male (*n* = 134)	Female (*n* = 84)	p-value
Registered Dietitians	25.23 (46)	22.22 (24)	23.53 (16)	35.71 (15)	0.216^a^	21.64 (29)	30.95 (26)	0.123^a^
Medical expert	13.76 (30)	8.33 (9)	16.18 (11)	23.81 (10)	**0.037**^**a**^	9.70 (13)	20.24 (17)	**0.028**^**a**^
Personal Trainer	20.64 (53)	21.30 (23)	22.06 (15)	16.68 (7)	0.772 ^a^	26.87 (36)	10.71 (9)	**0.004**^**a**^
Internet	42.20 (92)	50.00 (54)	36.76 (25)	30.95 (13)	0.058 ^a^	47.02 (63)	34.52 (29)	0.069 ^a^
Friends	26.61 (48)	32.41 (35)	23.53 (16)	16.67 (7)	0.116 ^a^	29.10 (39)	22.62 (19)	0.292 ^a^
Other Athletes	5.51 (12)	4.63 (5)	8.82 (6)	2.38 (1)	0.304 ^b^	7.46 (10)	2.38 (2)	0.109 ^b^
Family	5.96 (13)	6.48 (7)	2.94 (2)	9.52 (4)	0.365 ^b^	5.22 (7)	7.14 (6)	0.560 ^a^
Myself	3.67 (8)	0.93 (1)	4.41 (3)	9.52 (4)	**0.034**^**b**^	4.48 (6)	2.38 (2)	0.491 ^b^

Data are expressed as a percentage (n) for categorical variables. P-values for group comparisons were assessed by ^a^ Chi-squared Test, ^b^ Monte Carlo’s Test, or ^c^ Fisher’s exact test, as appropriate.

The age and biological sex do not seem to have any influence on monthly spending by consumers in FS (Table 6). Nevertheless, it was found that volunteers who bought FS to improve performance (*p* = 0.001) and increase energy (*p* = 0.004) spent more money than participants who bought it for health remaining **[data not shown]**.

**Table 6.** Analysis of data variables based on monthly expenditure on food supplements and sports foods.

	Total Population (*n* = 218)	Age				Sex		
Monthly expenditure on FS/SF		18-25 years old (*n* = 108)	26-40 years old (*n* = 68)	41-80 years old (*n* = 42)	p-value	Male (*n* = 134)	Female (*n* = 84)	p-value
≤20 €	35.78 (78)	37.03 (41)	41.18 (28)	23.81 (10)	0.397	35.82 (43)	35.71 (30)	0.789
21 - 50 €	42.66 (93)	43.52 (55)	38.24 (26)	47.62 (20)		44.03 (56)	40.48 (34)	
51 - 400 €	21.56 (55)	19.45 (21)	20.58 (14)	28.57 (12)		20.15 (27)	23.81 (20)	

Data are expressed as a percentage (n) for categorical or continuous variables. P-values for group comparisons were tested by Chi-squared or Monte Carlo’s tests, as appropriate. Abbreviations: FS, Food Supplements.

## 4. Discussion

In general, society has demonstrated a growing concern for both physical and mental health, which is evident in individuals’ day-to-day routines and choices [40]. In line with this, the first Portuguese sustainability survey revealed that over than half of the participants reported having a “health-conscious” diet, while 40.50 % affirmed engaging in regular exercise to improve their quality of life [41]. Furthermore, when individuals are in a gym setting, they tend to incorporate FS or SF into their routine [15,16]. Hence, the present research supports the evidence that gym-goers are large consumers of these products [14].

In the current analysis, a notable 71.95 % of gym-goers reported using at least one FS or SF, showing a higher adherence to supplementation than most of the studies previously conducted in Portugal, in which compliance ranged from 36 to 66 % [14,42,43]. Several factors can explain the discrepancy in these values. Firstly, the data collection took place in urban areas of Lisbon, regions where participants tend to possess higher financial stability, potentially contributing to increased consumption of these products [44]. Additionally, the variations in study protocols and consumers’ lack of knowledge about the definition of FS [45] may contribute to the observed differences. The methodology employed in our study, involving in-person interviews, may have influenced participants’ perceptions and the accuracy of reported FS consumption. In instances where participants self-report their data (e.g. online surveys), inconsistencies may arise, with individuals possibly failing to consistently categorize supplements accurately or opting not to include certain FS because they do not perceive them as such [45].

Although the evidence does not highlight biological sex as a determinant of consumption, in this analysis, men were significantly the main consumers, which is consistent with previous studies [27,38,43]. Consequently, these outcomes affected the significant differences in weight and height between consumers and non-consumers.

It was found that a significant number of gym-goers who consumed FS and SF had the main training goal of muscle growth. Conversely, non-consumers were mainly motivated by their well-being. Athletes looking to improve their performance or achieve specific hypertrophy goals can use FS to boost their results [11]. On the other hand, those whose main motivation for training is well-being prioritize physical exercise as a way of reducing daily stress or increasing quality of life [2]. However, it is important to mention that even those who go to the gym in search of well-being can use FS as a way to protect their health [6,7,17].

The data herein obtained also indicated that younger individuals were more likely to go to gyms motivated by hypertrophy and performance, while older individuals were mainly motivated by their overall well-being. Younger individuals often experience social pressures regarding appearance, which can influence their desire to increase muscle mass [2,3]. While older individuals typically start training to improve their physical and emotional well-being [46]. Promoting this practice is crucial particularly for seniors, to reduce the symptoms of many diseases lower the risk of fractures from falls, and foster independence [47].

When comparing sexes, it was found that men are more likely to go to the gym for performance improvement and hypertrophy, while women prioritize weight control and well-being. In modern society, there is growing social pressure that imposes specific beauty standards, such as valuing thinness as a “symbol” of feminine beauty and hypertrophy as an attribute of masculinity [2,3]. These social influences could lead individuals to join fitness centers just to feel like they fit into today’s beauty standards [2,3].

Exercise habits also seem to be a determining factor for FS consumption. There was a significant association between the use of these products and the number of training sessions per week. This association may be related to the fact that a greater number of training sessions can increase the macro-micronutrient needs of these athletes, leading to the consumption of FS to cover these needs [6,23]. Additionally, the gym environment itself can influence, as it often promotes the adoption of stereotyped routines, such as the usage of FS and SF [15,16]. Several studies have yielded comparable findings, reinforcing the idea that a higher frequency of training is associated with the consumption of FS and SF [27,37,38].

Regarding the preferred type of FS and SF consumed by gym-goers, the literature seems to be split between protein supplements [14,27] and MVMs [6,32]. In this study, the most ingested product was protein (59.17 %). This result aligns with the consumption range reported in previous studies, which varied between 12 and 74 % [27,42,48]. The preference for consuming protein supplements can be attributed to the numerous benefits associated with them [13]. Among the benefits are the positive impact on body composition, increased muscle mass, and improvements in endurance, performance, and strength [13]. The prevalent use of protein supplements can also be linked to the fact that it is not always feasible to receive sufficient amounts of protein solely from food, often due to constraints in preparation or time [14,49]. Consequently, the consumption of FS and SF is a convenient and effective alternative to ensure adequate protein intake [14]. In terms of age and gender differences, young people and male participants were more likely to use protein and creatine supplements. The main reason for this may be the aforementioned “beauty cult” [2,3]. Younger individuals and males are focused on achieving esthetic goals (muscle mass) [3], as also observed in the present analysis. Protein and creatine contribute to these specific goals, as they boost results in a shorter period [11]. While proteins are essential for growth and tissue repair [50], creatine seems to be beneficial for high-intensity and short-duration exercises [21,51].

Conversely, among older volunteers, we observed a different trend toward FS consumption. In this study, a higher percentage of older individuals prefer MVMs, omega-3, vitamin D, magnesium, and L-carnitine. These preferences are more aligned with overall health, highlighting a prevalent tendency among older individuals to opt for supplements that enhance their sense of well-being [2]. This trend for searching for well-being was also observed when older participants were asked for the main reason for training. These findings underline the importance of adapting supplement recommendations to the different age groups, considering their specific goals, and underscore the importance of guidance from healthcare professionals [52].

In contrast to males, it was found that women were more likely to consume protein yogurts. The active lifestyle that women often juggle (family, personal, and professional), makes snacking a convenient option [27,37,53], allowing them to meet the levels of protein required without the need to invest time in meal preparation [14].

The main reasons reported for beginning supplementation were muscle gain (57.34 %), health maintenance (41.75 %) and recovery (32.57 %). We also found that some of the reasons mentioned were strongly linked with consumers’ age group and biological sex. Several studies have also shown that increasing muscle mass is the predominant reason for consuming FS [27,42,54]. In those studies, it was also possible to observe a link between esthetic reasons and younger ages (< 40 years), as well as health reasons and older ages (> 40 years), corroborating the results of the current analysis. Additionally, women cited fatigue as one of the main reasons for FS and SF use, a trend potentially attributed to the higher incidence of particular deficiencies in this sex, such as anemia [55].

More than half of consumers reported to purchase their products online. However, the source of purchase differs according to the consumer’s gender and age. Younger consumers tend to opt for online purchases, being more acquainted with the digital realm and valuing convenience over security [56]. While the older ones appear to prioritize personalized service and the conventional approach of acquiring products from physical establishments [56]. Significant differences were also found between gender categories since women prefer pharmacies and physical stores, while men showed a preference for purchasing products from online sources. This is following previous scientific studies about the impact of gender and sources of purchase, in which women place more importance on assurance, an indicator related to privacy, security, and trust [57]. This may lead them to search for personal recommendations and face-to-face assistance, while men may feel enough confident in online shopping.

On the other hand, women’s preference for pharmacies and herbal shops may be related to the feeling of trust and security that these places can give [58].

When analyzing the sources of information was observed that the main sources were the internet, friends, and dietitians. These results corroborate the findings of other researchers [38,42]. However, a greater reliance on dietitians (≈ 25 %) was observed when compared to the majority of studies, in which only 10 to 19 % of participants reported using this health professional as a source of advice [27,42]. Women and older individuals significantly prefer doctors as a source of information. Conversely, men and younger individuals seem to prefer online sources, aligning with the findings obtained by El Khoury & Antoine-Jonville, in 2012 [42]. Thus, given the prevalence of less credible information sources, consumers need to be aware of the possible health risks and adverse effects associated with the consumption of FS [26,27]. Unlike pharmaceutical drugs, FS can be used without the need for oversight from a healthcare professional. However, it should be noted that sometimes health professionals may not have proper knowledge about FS and its legislation [59]. Furthermore, many FS are consumed without substantial scientific evidence to support their effectiveness [21,60]. Additionally, there is a lack of concern regarding the safety associated with the use of these products, which may not be guaranteed [21]. Consequently, there is apprehension related to inherent toxicity and the possibility of contamination or adulteration of FS, which could pose serious dangers to human health. Heavy metals [33], undeclared pharmaceuticals [61], as well as illegal compounds like doping [17], can all be present in FS.

The present study has shown that supplement consumption is high among gym-goers, in this way, individuals should be aware of the benefits and risks of supplements [52]. The implementation of programs that encourage consumer awareness of recommended dosages, interactions between substances, and possible adverse effects should be paramount [52]. Health professionals must take primary responsibility for providing this guidance for consumers to be able to make safe decisions [52].

### 4.1. Strengths and limitations

One of the strengths of this study is that provides further evidence on the consumption of supplements by Portuguese gym-goers, despite being a pilot study performed in the Lisbon Metropolitan area. There is very limited evidence on this subject, raising some questions about market analysis and athletes’ knowledge of supplementation. However, several limitations must be acknowledged, like the cross-sectional design, convenience sampling, and small sample size do not allow a causal effect to be established. The fact that height and weight were self-reported could also be considered a limitation because the general population tends to overestimate height and underestimate weight, which may have an impact on BMI results from [62], no analysis by BMI was performed.

## 5. Conclusion

The consumption of FS/SF is widespread among gym-goers in Lisbon (Portugal). The most consumed FS/SF in the analyzed population were protein (including bars, shakes, and yogurts), amino acids, caffeine, and MVM. Protein was more consumed by younger males, and MVM and creatine by older people and women. These results were strongly connected with fitness goals and physical objectives since it was observed that men and younger consumers were more likely to use products related to hypertrophy and performance enhancement. At the same time, women and older focused more on health and well-being.

Gym-goers rely on various sources for supplement information, including the internet, friends, dietitians, personal trainers, pharmacies, and medical doctors, differing these sources among age ranges and gender.

These findings suggest a need for targeted guidance and education, considering the higher consumption, fitness motivations, sources of information, and preferences among different demographic groups.
